# Supplementary material for: Predicting visual function by interpreting a neuronal wiring diagram
Source: Nature. 2024 Oct 2;634(8032):113–23. doi: 10.1038/s41586-024-07953-5 (PMC11446822; doi:10.1038/s41586-024-07953-5)
Supplement: Supplementary file 6 — CRF and ERF predictions for individual TmY4 and TmY9 cells. Analogous to Supplementary Data 3, but for TmY target types. Shown are the top four monosynaptic pathways, the strongest pathway passing through each of the top ten intermediary types (ranking from Extended Data Fig. 7), and the trisynaptic pathway Tm1–TmY–Dm3–TmY (see the section entitled Prediction of spatial normalization). [file 41586_2024_7953_MOESM6_ESM.zip › DataS4/TmY9q/720575940631961960.pdf]

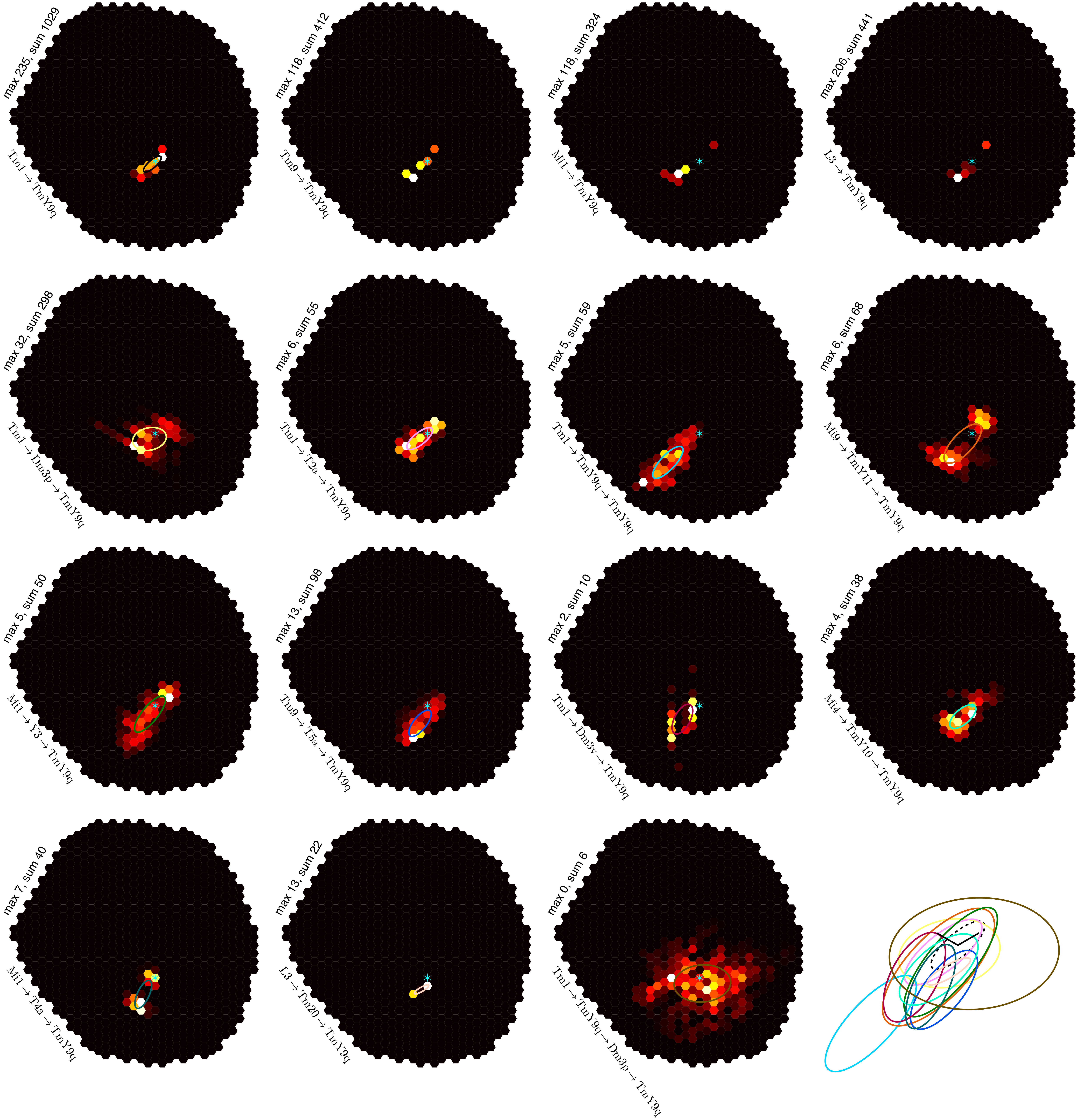

max 235, sum 1029
Tm1 → TmY9q
max 118, sum 412
Tm9 → TmY9q
max 118, sum 324
Mi1 → TmY9q
max 206, sum 441
L3 → TmY9q
max 32, sum 298
Tm1 → Dm3p → TmY9q
max 6, sum 55
Tm1 → T2a → TmY9q
max 5, sum 59
Tm1 → TmY9q → TmY9q
max 6, sum 68
Mi9 → TmY11 → TmY9q
max 5, sum 50
Mi1 → Y3 → TmY9q
max 13, sum 98
Tm9 → T5a → TmY9q
max 2, sum 10
Tm1 → Dm3v → TmY9q
max 4, sum 38
Mi4 → TmY10 → TmY9q
max 7, sum 40
Mi1 → T4a → TmY9q
max 13, sum 22
L3 → Tm20 → TmY9q
max 0, sum 6
Tm1 → TmY9q → Dm3p → TmY9q
